# Optometry preceptors’ perceptions of clinical supervision at a South African university

**DOI:** 10.4102/hsag.v30i0.2836

**Published:** 2025-06-11

**Authors:** Zamadonda N. Xulu-Kasaba, Firdous B. Hoosen, Tina Mlanji, Naadira Moosa, Sthembile Ngcobo, Idani B. Nelwamondo, Olivia B. Baloyi

**Affiliations:** 1 Department of Optometry, Faculty of Health Sciences, University of KwaZulu-Natal, Durban, South Africa

**Keywords:** optometry, preceptorship, clinical supervision, health professions education, undergraduate clinical training, South Africa, clinical teaching effectiveness, clinical training, university clinics, qualitative research

## Abstract

**Background:**

Effective clinical teaching in tertiary institutions relies heavily on clinical preceptors who supervise undergraduate students. While many health science disciplines have explored clinical supervisors’ perceptions of their roles, limited evidence exists in optometry.

**Aim:**

This study aimed to explore optometry clinical supervisors’ views on their effectiveness as clinical teachers.

**Setting:**

One-on-one interviews were conducted online via Zoom.

**Methods:**

Interviews were conducted in English and transcribed verbatim. Using a social constructivist paradigm and the inductive approach of content analysis, the data were analysed. Twelve of 22 eligible clinical supervisors (aged 21 years –50 years; 75% female) participated in the study.

**Results:**

Two main categories emerged: (1) Roles and responsibilities of optometry preceptors, and (2) Intervening conditions that facilitate or hinder effective supervision. Participants described the clinic as a nurturing learning environment, with supervisors modelling professional behaviour, prioritising learning, and fostering psychological safety. Supervisors supported students in developing higher-order thinking. Facilitating factors included collaboration with academic staff and institutional support. However, high workloads, lack of adequate remuneration, and human resource challenges were identified as barriers to effective supervision.

**Conclusion:**

Optometry clinical supervisors were committed to delivering effective preceptorship and creating a supportive clinical learning environment. Addressing workload, remuneration, and administrative challenges could enhance the supervision experience and sustain high-quality clinical teaching.

**Contribution:**

This study identifies key enablers and barriers to effective clinical supervision, offering insights to improve the clinical training experience for undergraduate optometry students.

## Introduction

Clinical education has been defined as the provision of guidance and feedback on personal, professional and educational development in a trainee’s experience of providing appropriate patient care (Kilminster et al. 2007). As such, clinical education is important for the development of health professionals, like optometrists, and for providing quality patient care (Grant et al. 2003; Kilminster et al. 2007). Clinical training is a significant and unique component of healthcare education, as is the supervisory process that goes along with it (Strohschein, Hagler & May 2002). Several authors agree that clinical education is important for the development of professional skills and that it is an essential element of the healthcare educational programme (Chan 2001; Strohschein et al. 2002). As students learn within the context of clinical practice, the clinical learning setting is ideally suited to support the development of professional skills (Strohschein et al. 2002).

The didactic clinical supervisory process and clinical education are two significant and important components in the training of health care professionals (Strohschein et al. 2002). In the clinical learning experience, theory consolidates into practice, and students learn to combine and integrate the knowledge, skills, attitudes, values and philosophies of the profession under the guidance, and attributes, of a clinical supervisor (Chan 2001; World Health Organization [WHO] 2022). The clinical learning environment is the ideal area in which to facilitate professional skills, ensure patient safety and promote high clinical standards, as students are learning within the real-life context of clinical practice (Grant et al. 2003; Levy et al. 2009; Strohschein et al. 2002). The WHO Eye Care Competency Framework (ECCF) identifies six key competency areas in which optometry students must demonstrate proficiency before completing their undergraduate training (WHO 2022).

Clinical teaching is an essential component in the education of undergraduate optometry students and is pivotal for transferring knowledge into practice (World Council of Optometry 2015b). Within the South African context, the first 2 years of the optometry degree *include* clinical technique training in laboratory-based settings. In this period, students engage in simulated learning, where they practice foundational skills such as case history approaches, preliminary tests, basic clinical techniques and basic refraction under simulated, supervised conditions. This laboratory-based phase enables students to gain theoretical knowledge and practical expertise *before* transitioning to real-life clinical settings and real patients. In the second half of the degree, clinical training takes place in patient-based clinics, where students apply their knowledge and skills in actual patient care, helping them improve preparedness and reduce anxiety as they progress *towards* professional practice (Chen et al. 2023; Natesan et al. 2020; World Council of Optometry 2015a, 2015b, 2024). To achieve effective clinical teaching and learning experiences, training institutions rely on experienced and competent optometrists, engaging directly in the field, to function as clinical supervisors to guide and assist students. In addition, clinical supervisors monitor student optometrists’ ethical *behaviour*, ensure safe *people-centred* practice and enable learning and translation of didactic information into a clinical environment (Levy et al. 2009; Strohschein et al. 2002).

Previous studies have investigated the importance of clinical education for professional skill development. In various healthcare fields, clinical supervision is perceived as a crucial educational task that acts as a bridge for active clinical learning. Clinical supervisors have affirmed that their effectiveness in these roles is demonstrated through their responsibilities as tutors, leaders and role models for students (Girotto et al. 2019). Effective clinical supervision is enhanced when students not only apply theoretical knowledge gained in lectures but further actively prepare for real-world clinical experiences. This preparation ensures the integration of theory with practice, improving their readiness to handle clinical challenges and providing a solid foundation for patient care (Chen et al. 2023; Natesan et al. 2020). In addition, clinical supervisors affirmed that they bear the responsibility of enhancing students’ learning processes (Levy et al. 2009). In optometry, however, previous studies limited their investigations to the clinical supervisors’ perceptions of decentralised training sites’ infrastructural ability to cater to optometry students’ clinical education (Kirkman et al. 2022). This study aimed to explore and describe optometry clinical supervisors’ perceptions of their effectiveness and role in ensuring positive clinical accompaniment for optometry students.

## Research methods and design

### Research paradigm

This research study was underpinned by a social constructivism paradigm because the researchers relied on the participant’s views, practices and processes of the situation being studied (Cresswell & Poth 2018; Crotty 1998). The researchers acknowledged that the views, practices and processes of the optometry clinical supervisors on preceptorship were of utmost importance in this study. The three main points of social constructivists were adhered to, namely: (1) as humans we construct meaning through our interaction with the world around us, (2) cultural perspectives play an important role in how we understand and interpret the world around us, (3) any meanings we generate are based on social interactions in the community (Elo & Kyngäs 2008).

The research design determined *how* the research was conducted (Lincoln & Guba 1985). The study was qualitative, exploratory, contextual and descriptive. An exploratory design was used in this study because there was evidence of a paucity of literature on perceptions of optometry preceptors on preceptorship, particularly at the study university. Furthermore, the study aimed to explore the phenomena from the viewpoint of the participants as they had knowledge and experience *in* preceptorship. The exploratory nature of this study also aided the researchers *in gaining* in-depth insight and understanding regarding the perception of optometry preceptors about preceptorship.

### Recruitment of participants

All eligible participants, including the two selected for pilot interviews, were known to the research team. A Google Form link was emailed to all potential preceptors as an invitation to participate in the study. Thereafter, in keeping with Section 18 of the *Protection of Personal Information Act* (POPIA), the research team agreed on one researcher who would conduct data collection. The researcher approached potential participants face-to-face in an attempt to seek their voluntary participation in the study. An information sheet was given to the potential participants explaining the study. Ethical issues relating to this study; purpose and data-collection method; were explained and any questions from the participants were answered. Both the researcher and potential participants who expressed their interest *in participating* in the study agreed on a data-collection date and time. All potential participants voluntarily shared their mobile phone numbers and email addresses with the researcher to facilitate data-collection arrangements.

### Population, sampling, inclusion and exclusion criteria

To ensure appropriate and useful information that would yield rich data, adequately address the study’s aim and improve trustworthiness; purposive sampling was employed in this study (Elo & Kyngäs 2008). In all, 22 *preceptors* who were involved with the clinical supervision of optometry students at the institution were approached. A total of 12 optometry preceptors agreed to participate in the study. The participants affirmed that they were willing to participate voluntarily. The researchers explained that the participants would be contacted individually and that interviewing would continue until data saturation was reached. Further, 10 clinical supervisors who did not indicate an interest to participate in the study were excluded.

#### Study setting

Data enrolment and collection took place at the university’s eye clinic. For participants who could only be interviewed after hours, interviews *were conducted* through an online meeting using the Zoom platform.

#### Data-collection tools and procedures

In qualitative research, the data-collection tool is mainly the researcher himself or herself. In this study, data were collected by one member from the research team. Online in-depth interviews were held during July and August 2023. The co-facilitator assisted with interviewing, and writing field notes, and acted as a timekeeper; this allowed the researcher to focus and concentrate on the interview. The piloted interviews were tape-recorded and transcribed verbatim. The findings and transcripts were shared with the supervisor, who has expertise in optometry both in academia and clinically. Making use of the experience of the supervisor and co-supervisor, additional coaching and recommendations in terms of how to probe deeper were offered. These trial interviews were not included in the analysis.

An interview guide had one open-ended question ‘As an optometry preceptor what are your perceptions about your role in ensuring positive clinical accompaniment for optometry students’. The following probes were used to elicit more data from the participants or to seek clarity: ‘tell me a bit more’ and ‘thank you very much, is this what you meant’. To ensure all the participants were familiar with the phenomenon being studied, the researcher defined any unfamiliar concepts to the participants before and during the interview; this ensured the participants followed the discussions. The interviews were tape-recorded and conducted in English, which was the language preferred by the participants. All interviews lasted between 30 min and 45 min and were tape-recorded and transcribed verbatim. At the end of each interview, the researcher summarised the core points and issues, which were discussed, and each participant was allowed to add any final comments. The summarised core points were then compared with the field notes and kept safe to provide a system of backup information throughout the data collection.

### Data-collection process

All ethical approvals were obtained *before* data collection. The researcher proceeded by welcoming the participants to the session and acknowledged and reassured them of the privacy and confidentiality of the session. Furthermore, the researcher delineated online participation etiquette, inclusive of the preservation of privacy (participants were advised to turn off their cameras during the session, which could furthermore minimise possible extra data charges). The information letter was read clearly and loudly by the researcher. Following *this*, the participant was given time to internalise the contents of the letter. *The participant* was then encouraged to raise questions and concerns for clarity. The researcher confirmed that the participant had signed the consent form to participate in the study and for the digital recording, *before* the commencement of the interview sessions. The tenth interview evidenced data saturation and the last two interviews served to ensure that no new information is emerging. After the tenth interview, it was decided that as there was no new information coming through, data saturation had been reached. An additional two interviews were conducted to ascertain data saturation and redundancy. As such, the final sample size was concluded as *N* = 12.

#### Data analysis

The Inductive Approach of Content Analysis, by Elo and Kyngas, was used to analyse verbatim transcribed in-depth individual interviews (Elo & Kyngäs 2008) This is a systematic technique for compressing many words of text into fewer content categories based on explicit rules of coding. The content analysis processes are categorised into three main phases; Preparation, Organising and Reporting (Elo & Kyngäs 2008).

### Organising and preparation of data

Two recorders were used to collect data, *to* ensure audibility and accuracy for the transcription. All interviews were transcribed verbatim, line-by-line and *word-for-word* by the researcher. Self-chosen pseudonyms were used to identify the participants, ensure anonymity and enable researchers to use direct quotations to enhance the credibility of the discussion and findings. Field notes were read while listening to the recordings in an attempt to ensure the consistency of the data. To develop a general sense, as the researcher transcribed the data, she listened repeatedly to the recorded data. Re-reading of data was done across all interviews, allowing for the development of a general sense of the data collected and transcribed. In addition, the researcher was able to reflect on its meaning; and add further underlying meanings into the analysis notes simultaneously. Following transcription, a soft copy of data was stored electronically on NVIVO version 14, on one of the team’s computers; thereafter hard copies of data were printed.

### Coding of the data

The research question and the study objectives were reviewed. The participant’s perceptions of their role in ensuring positive clinical accompaniment for optometry students were noted and highlighted across all individual interviews. Perceptions that occurred frequently across the individual interviews were noted and underlined, forming text segments. A phrase was assigned that best described the text segment, forming codes. Most descriptive wording *was* used by the researcher to give a topic for all similar codes to form categories. The related codes were grouped and sub-categories were identified in each category where appropriate. These categories were then *analysed* for each interview. The multiple perceptions received from different respondents and each interview were identified and described using participants’ quotations, which in turn described and explained each phenomenon in more detail, and increased the trustworthiness of the data. Independent data coding was done by the researcher together with the research supervisor and co-supervisor to ascertain consensus about the developed categories and sub-categories.

### Representing the findings

A summary of findings, categories and sub-categories were represented in a table form. Initially six categories emerged based on meaningful units within the text. However, with further data reduction these were finally condensed into two categories. The supervisor and co-supervisor, who are also the co-authors in this article were involved, throughout the whole process of analysis. For consensus to be reached, the coding and analyses were discussed between the three several times. Verbatim quotes from the participants were selected to strengthen and ensure transparency for the choice of categories and sub-categories. An integration of the literature review and findings was done to extend and explain the categories and add to the richness of the findings. Interpretation of the data was according to (Cresswell & Poth 2018) the interpretation stage involves making sense of the data through reflection, including the researcher’s personal views and comparing new findings with past studies (Cresswell & Poth 2018). The overall meanings of the findings will be discussed in detail.

#### Trustworthiness

Credibility focuses on consistency in the formulation and interpretation of available data. Credibility ensures that the findings are based on the data (Cresswell & Poth 2018; Elo & Kyngäs 2008). In keeping with investigator triangulation, data were independently coded, analysed and interpreted by three researchers. This was in addition to participants’ member checking, where data were returned to participants to ascertain accuracy (15). Transferability is the extent to which the study findings can be transferred to other settings or groups (Cresswell & Poth 2018; Elo & Kyngäs 2008). Transferability was achieved through provision of a thick description of the research procedures, study context and setting and findings. Further to this, the study involved optometry preceptors with varied demographics (age, years of experience, qualifications and gender) ensuring a dense description of the population studied (Lincoln & Guba 1985).

Dependability is the method concerned with the stability of data in the study (Polit & Beck 2017). To allow for dependability interviews were transcribed immediately and the supervisors closely monitored the data analysis process through regular debriefing meetings with the researcher (Polit & Beck 2017). In addition, a thick description of the study methodology and data was provided. Confirmability occurred once credibility, transferability and dependability were established. Throughout the data-collection process, the researcher avoided leading and determining the direction of interviews, instead participants were asked for clarification whenever needed. The researcher created an audit trail to ensure the data’s accuracy, relevance and meaning. This included unstructured interviews that were audio-recorded and transcribed verbatim, with written field notes.

#### Ethical considerations

Permission to conduct the study was sought from all relevant authorities. Full ethical clearance was obtained from the University of KwaZulu-Natal, Biomedical Research Ethics Administration (BREC) (reference no.: BREC/00005579/2023) and gatekeeper permission from the registrar of the participating university. In accordance with standards set by the university’s research committee, participants’ rights were respected throughout the study.

Participants were provided with an information sheet detailing their rights, the research aim, the methodology and an opportunity to provide voluntary written consent. Participants’ rights to decline participation in the in-depth individual interviews inclusive of their rights to withdraw at any given stage of the research were discussed. The names of the researcher and supervisors and the research ethics committee were available on the information sheet in case of any queries.

Aligned with Sugiura et al. to ensure confidentiality, the information supplied by the clinical supervisors. through individual interviews was not linked to their names as they were not addressed by their real names during the interview (Sugiura et al. 2017). Instead, self-selected pseudonyms were used. The audio-recorded interviews were accessible only to the researcher and her supervisors. All research data, including the audio, were stored in a lockable filing cabinet in the supervisor’s office within the university and will be destroyed after 5 years. Justice: All optometry preceptors employed at the study setting during data collection had an equal chance to participate without any coercion (Sugiura et al. 2017). Respect for autonomy: Participants were aware that their participation in the individual interview was voluntary and that they could withdraw from the study without any consequences at any time if they so wished (Sugiura et al. 2017). Clinical supervisors were not coerced or pressured to participate in the study. Risk of harm: No physical risks were anticipated, the researcher reassured the participants.

## Results

### Profile of the participants

A total of 12 individual interviews were performed with optometry preceptors. Details on the profile of the participants, using their pseudonyms, are shown in Table 1. Various themes and sub-themes (as shown in Table 2) emerged from the study.

**TABLE 1:** Profile of participants (*N* =12).

Participants	Pseudonym	Gender	Experience
P1	Beige	Male	Less than 3 years
P2	Ivory	Male	3–6 years
P3	Amber	Female	less than 3 years
P4	Olive	Female	3–6 years
P5	Maroon	Male	Less than 3 years
P6	Teal	Female	more than 6 years
P7	Indigo	Female	more than 6 years
P8	Burgundy	Female	3–6 years
P9	Orange	Female	more than 6 years
P10	Cream	Female	3–6 years
P11	Turquoise	Female	less than 3 years
P12	Bronze	Female	3–6 years

**TABLE 2:** Categories and sub-categories.

Category	Sub-category
1. Roles and responsibilities of an optometry preceptor	1.1. Bridge the gap between the classroom and the clinical area 1.2. Serve as a professional role model to optometry undergraduate students 1.3. Creating nurturing clinical learning environment
2. Intervening conditions	2.1. Facilitative factors for effective preceptorship 2.2. Hindering factors for effective preceptorship

#### Theme 1: Roles and responsibilities of an optometry preceptor

Optometry clinical supervisors in this study perceived themselves as having a tremendous task to ensure effective and efficient clinical mentoring and support to undergraduate optometry students. To equip these individuals to be relevant and responsive to the diverse needs of their patients and the communities they would later work in. The following extracts supported this statement:

‘I also help the students to understand the patient setting that they’re going to go into, and how they must conduct themselves, for one. Two: how they must tie in case history with the final clinical decision, and three, if they want to keep that patient to keep on coming to them regularly, the kind of skills they need other than academic or optometry. They need compassion, and they need a relationship with their patients.’ (P5)

**Bridge the gap between the classroom and the clinical area:** All the participants in this study perceived their role in bridging the theory-practice gap, as key in ensuring a positive clinical learning experience for optometry students. Quotes by participants relating to this statement are as follows:

‘I look at the notes ahead of going to the clinic and I try and understand them from the perspective that they were delivered on. Then I try and implement that as combined knowledge where I take bits and pieces of the theory, and I add it into the technique to try and create an understanding of where this technique is placed, why it’s done, how it’s done, and what’s the scientific basis for it.’ (P8)

‘Oh, yes, yes, they they do. The clinics are an integral part of the teaching. They can’t just go through getting knowledge without doing the clinics.’ (P10)

‘During covid it was difficult obviously, but the more outside patients they get, the better the exposure to real patients, the better it is for their training. So the whole going to hospitals uh, all of that helps it really does.’ (P12)

**Serve as a professional role model to undergraduate optometry students:** In their attempt to enhancing a positive clinical experience for optometry students, the participants felt that serving as professional role models is of utmost importance. The following narratives attest to this statement:

‘A preceptor is more of a role model. Students come from different backgrounds, and they may respond in ways that aren’t always pleasant.’ (P11)

‘But as a preceptor, who is a role model shouldn’t judge them based on their actions before or outside of the clinic.’ (P8)

‘The focus should be on how they performed that day and correct them when needed.’ (P11)

‘Like a role model, even if a student makes a mistake or doesn’t respond well, you don’t hold it against them. You guide them, help them learn, and remember that they are still learning.’ (P10)

‘By the time I’ve worked with a student and helped them improve, I can feel proud of the progress they’ve made. Being a role model, guiding and helping students grow, is one of the key qualities of being a good preceptor.’ (P11)

‘There is so much of work. As a student you can’t say the teacher is the teacher, you really need to have a relationship with the teacher and be comfortable for you to be able to go ask questions without being scared.’ (P2)

So students are less scared of you, they are more welcoming of you so they are gonna slightly be more on the comfortable side when they can ask you questions anytime. Not that they will just be waiting for you to leave the room so that they can be free.’ (P2)

**Creating a nurturing clinical learning environment:** Creation of a nurturing clinical learning environment was mentioned by all the data sources as the core to enhancing a positive clinical environment. According to them, a nurturing clinical learning environment is not only effective for clinical learning, but such environments are psychologically and intellectually safe. They enhance students’ development of higher order thinking skills. Participants verbalised this as follows:

‘A preceptor should also be someone who doesn’t think teaching is about shouting.’ (P3)

‘Someone who has the patience, uhm I think for me. I think I’ve learnt from myself: when you shout I block everything. I like block my ears, but I am looking at you and it’s as if I am listening. So they should have this quality of that even if you want to shout, or maybe the student is just not understanding what you’re saying and you feel agitated or you were having a bad day, either way try and keep it professional.’ (P2)

‘Try and make sure that you’re always making them feel welcome. As much as you don’t want them to cross the line of being too friendly, but try and not shout too much so that the student will be able to learn from their mistake.’ (P6)

‘If someone makes a mistake, then all you do is shout: “what are you doing, this is wrong stupid!!”, you know, that person is never going to be able to learn what they are supposed to do right. The next thing, since you said they are stupid, they will just shut off. So less shouting, more being polite, I guess.’ (P6)

‘My most important thing, I think from the time I’ve started to now, is understanding students and the emotional background they’re coming from. If you can counsel them when they’re going through financial situations or they’re going through family situations, to being a little bit more compassionate. I also use that as a building block to building a relationship with them.’ (P10)

#### Theme 2: Intervening conditions

Apart from their roles and responsibilities, participants mentioned that there were factors that either contributed positively or negatively in ensuring positive clinical learning for undergraduate optometry students.

**Facilitative factors for effective preceptorship:** Collaborative teamwork between classroom lecturers, clinical optometrists and preceptors emerged as facilitative factors for effective preceptorship. According to the data sources, collaborative teamwork enabled the smooth shaping of undergraduate optometry students in becoming fully fletched optometrists. The following quotations give evidence that collaborative teamwork enhances effective preceptorship for undergraduate optometry students:

‘Yes, they were. Yes, they were in a sense that they were obviously practical manuals which did help, sort of structure, the content that was delivered. So yeah, the answer is yes. Almost definitely yes. That was my experience. The lecturers were very much forthcoming and supportive in, in terms of that. Yeah. When, when the senior management works hand in hand with the junior staff-so preceptors- it, it does all in, all assist in in, in, in, in, in achieving the, required mandate.’ (P9)

‘I think that the modules that I worked in, the co-ordinators of those modules were very efficient people. They had a good setup for what they expected from us and they provided us with good resource and materials support, and that made it a very pleasant experience, and I think that the mix of preceptors in the clinics that I’ve worked with were really nice.’ (P3)

‘We all uhm we we are not previously exposed to each other or or socialised in the same spaces, but we uhm we we all had personality types that allowed for healthy and safe engagement, and I think that was beneficial to us and to the students as well. There was no conflict, you know, between preceptors at any time.’ (P3)

**Hindering factors for effective preceptorship:** The perceived hindering factors for effective preceptorship according to the data sources were time pressure and productivity demands. Participants highlighted the fact that time pressure and productivity demands predisposed them to working long hours which impacted negatively on their ability to make preceptorship memorable for the students. The remuneration offered for this was not ideal as it was not even near the market rate. The following extracts provide evidence to this statement:

‘In the clinic, sometimes I’ll admit, it’s frustrating. The admin side of it is very frustrating as you have to re-apply for your post every semester. We we don’t get paid in the month we apply in; we work for one month, submit a claim, and get paid by the end of the next month. So it’s difficult to recommend [*the preceptorship posts*] to people who are not in this whole teaching thing.’ (P7)

‘It’s difficult when you tell other optoms to come and work at the campus clinic, they literally laugh in your face. They say “you can do that, we need money at the end of the month”.’ (P5)

‘There hasn’t been a raise in the last six years! We’re working at the same rate every year while the cost of living is going up everywhere else, but if you bring that up, it seems like management, not optometry, seems like higher up management, just doesn’t care.’ (P5)

‘So it’s difficult to maintain that level of motivation to keep coming back. Eventually people are like “I’m fed up, I don’t need this in my life, I’d rather work somewhere I’m appreciated.” Often you feel like there’s a lack of appreciation, if that’s the word. It’s like it’s the same without you anyway.’ (P7)

‘The recommendation is that preceptors, given the fact that they not only supervising they’re also teaching, get standardized training, that’s a key take home message. But I also think that the amount of money that gets paid per hour, the [*amount in ZAR*] per hour, is honestly an insult for the time, the skills and the input that is required from the preceptors.’ (P12)

‘I think that [*amount in ZAR*] an hour in a regulated industry uhm, where you’re teaching other clinicians who are going to manage patients in real life uhm, I think [*amount in ZAR*] is an insult. It really needs to be looked at more realistically, that’s not even the cost of petrol for the day. Passion is unfortunately not enough to just pay the bills you know. So as much as preceptors have to be passionate, they need to get paid and the salaries that they are currently offering [*to optometry clinical preceptors*] at this institution is an insult.’ (P4)

## Discussion

The study findings highlighted three roles and responsibilities of optometry preceptors in ensuring a positive clinical learning experience for optometry students. *Bridging the Theory-Practice Gap*: In agreement with Myrick and Yonge the study participants perceived their preceptorship role of bridging the theory practice gap, as key in ensuring a positive and nurturing clinical learning environment for optometry students, whilst ensuring a significant increase in their clinical competence and confidence (Myrick & Yonge 2001). *Creating Nurturing Clinical Learning Environment*: Nurturing clinical learning environments emerged in the study as not only effective for clinical learning but such environments are psychologically and intellectually safe thus enhancing, in the students, the development of higher order thinking skills (HOTS). According to them (clinical supervisors), effective and efficient clinical preceptorship is not only about teaching the students, in addition it is to further ensure that students embrace professional conduct and ethical norms. *Serve as a Professional Role Model to Optometry Undergraduate Students*: Henceforth, the optometry preceptors perceived their professional role-modelling as a pivotal task. This particular study finding is in line with findings in previous studies, which reported that supervisors are key in role-modelling to clinical students, particularly through mentoring these emerging professionals to become responsible practitioners (Girotto et al. 2019).

Preceptors themselves add to the professional image that is part of the optometry profession as they are key to moulding the students they teach. As such, preceptors in addition bear the responsibility of ensuring that their conduct enables a pleasant working environment for each student in their future. Skills imparted here further equip students to learn to think quickly and efficiently, as well as the ability to make well-processed clinical decisions under pressure; a clear advantage for a knowledgeable eye health workforce in future practice (Xulu-Kasaba, Mashige & Naidoo 2021).

Facilitative Factors for Effective Preceptorship: As much as the clinical preceptors were aware of their roles and responsibilities, they had no issues executing certain facilitative factors that emerged as necessary for effective preceptorship. According to them, collaboration between academic and clinical teaching makes the preceptorship role more enjoyable through the strengthening of optometry clinical and theoretical education. In agreement with studies by Baloyi et al., together with Bvumbwe et al., a collaborative culture between the academic and clinical spaces ensures that students receive quality supportive education, unified by a combination of expertise from both academic knowledge and clinical competence. Furthermore, such collaborative approaches improve clinical personnel’s attitudes towards students and academics as it fosters feelings of mutual respect and affirms that both are needed in student development (Baloyi & Mtshali 2018; Bvumbwe 2016). Baloyi et al. further affirm that appreciation of clinical personnel’s clinical teaching role through collaboration has the potential to bridge the theory practice gap (Baloyi & Mtshali 2018).

In addition, sharing of teaching resources, and a discussion on clinical concepts prior to beginning a laboratory-based skills teaching session, was seen as an enabler to positive preceptorship as it emphasised the value of the clinical preceptors. In agreement, Myrick and Yonge affirmed that this kind of engagement reassured clinical preceptors of their importance as collaborators in the academic’s teaching programme (Girotto et al. 2019; Myrick & Yonge 2001).

Employees who feel that they are valued by their employers are likely to make every effort to surpass the call of duty (Gabris & Mitchell 1985). In this study, it was encouraging to see that clinical supervisors in the optometry clinic were validated by the positive collaboration they shared with the academics within the faculty. The results of this can only enrich student clinical teaching.

Hindering Factors for Effective Preceptorship: On the contrary, participants were concerned about the high workloads paired up with a remuneration that was below the market rate. These impacted negatively on their satisfaction levels, as they translated to unremunerated long working hours. As such, these factors negatively affected preceptors’ willingness to dedicate one-on-one individualised attention to students in clinical training sessions. These hindrances were challenging as they highlighted the lack of support by managers who were tasked with ensuring adequate staffing numbers for optimal supervision ratios under satisfactory working conditions. As such, in agreement with previous studies, it confirmed that the clinical supervision role is difficult when there is inadequate support from the management in a healthcare environment with a high workload (Girotto et al. 2019). In addition, various authors expressed that the burden of a high workload in poorly resourced institutions ultimately overwhelms clinical supervisors (Burgess, Van Diggele & Mellis 2018; Byrd, Hood & Youtsey 1997). This in turn could limit the mentorship relationship that supervisors are able to offer clinical students, and negatively impact the enjoyable preceptorship experience for the students (Byrd et al. 1997; Latessa et al. 2019).

In light of this, it might be worthwhile to address these concerns in an attempt to improving the preceptorship environment overall. Optometry clinical supervisors were dedicated to facilitating the clinical training of students. To ensure a memorable clinical learning experience, they created an optimal clinical learning environment, establishing a psychologically and intellectually secure environment for students. As students’ role models, they encouraged students’ critical thinking and higher order thinking skills. Close collaboration between lecturers, clinical preceptors and students facilitated the learning environment. A high workload, productivity demand and remuneration issues were mentioned as hindering factors to effective preceptorship.

### Limitations of study

Conducting the study at one institution was a limitation as clinical supervision is an integral part of training health professionals. As such, it would be beneficial to conduct this study at more than one training institution as it would give a broader view of this matter and improve the sample size. Further to this, the absence of student voices is a limitation that should be addressed in another study.

### Recommendations

Aligned with the study findings, the authors of this article recommend that training institutions should enhance partnerships among academics and clinical preceptors to bridge the theory–practice gap. Frequent meetings, joint training sessions and shared teaching resources can improve the alignment of theoretical and clinical training.

## Conclusion

The current findings suggest that preceptors, as role models, play a vital role in encouraging optometry students to develop higher-order thinking skills such as critical thinking skills. The close collaboration between optometry lecturers, clinical preceptors and students emerged from the study as a crucial factor in enhancing a conducive clinical learning environment. However, it is worth noting that factors such as unmanageable workloads and the need for market-related remuneration rates emerged as potential hindrances to effective preceptorship. These factors should be carefully managed to ensure a supportive and effective preceptorship experience, as they have the potential to hinder the overall success of the learning process.
